# Enhanced Recovery After Pancreatic Surgery Does One Size Really Fit All? A Clinical Score to Predict the Failure of an Enhanced Recovery Protocol After Pancreaticoduodenectomy

**DOI:** 10.1007/s00268-020-05693-x

**Published:** 2020-07-30

**Authors:** Giovanni Capretti, Marco Cereda, Francesca Gavazzi, Fara Uccelli, Cristina Ridolfi, Gennaro Nappo, Greta Donisi, Andrea Evangelista, Alessandro Zerbi

**Affiliations:** 1 Pancreatic Surgery Unit, Humanitas Clinical and Research Center – IRCCS, via Manzoni 56, 20089 Rozzano, Milan, Italy; 2 School of Medicine and Surgery, University of Milano-Bicocca, San Gerardo Hospital via Pergolesi 33, 20900 Monza, Italy; 3 Department of Biomedical Sciences, Humanitas University, Via Rita Levi Montalcini 4, 20090 Pieve Emanuele, Milan, Italy; 4 Department of Economy and Statistic, University of Torino, Torino, Italy

## Abstract

**Background:**

The inability to comply with enhanced recovery protocols (ERp) after pancreaticoduodenectomy (PD) is a real but understated issue. Our goal is to report our experience and a potential tool to predict ERp failure in order to better characterize this problem.

**Methods:**

From January 1, 2014, to January 31, 2016, 205 consecutive patients underwent PD in our center and were managed according to an ERp. Failure to comply with postoperative protocol items was defined as any of: no active ambulation on postoperative day 1 (POD1); less than 4 h out of bed on POD2; removal of nasogastric tube and bladder catheter after POD1 and POD3, respectively; reintroduction of oral feeding after POD4; and continuation of intravenous infusions after POD4. Data were collected in a prospective database.

**Results:**

Taking in consideration the number of failed items and the length of stay, we defined failure of the ERp as no compliance to two or more items. A total of 116 patients (56.6%) met this definition of failure. We created a predictive model consisting of age, BMI, operative time, and pancreatic stump consistency. These variables were independent predictors of failure (OR 1.03 [1.001–1.06] *p* = 0.01; OR 1.11 [1.01–1.22] *p* = 0.03; OR 1.004 [1.001–1.009] *p* = 0.02 and OR 2.89 [1.48–5.67] *p* = 0.002, respectively). Patient final score predicted the failure of the ERp with an area under the ROC curve of 0.747.

**Conclusions:**

It seems to be possible to predict ERp failure after PD. Patients at high risk of failure may benefit more from a specific ERp.

## Introduction

Pancreaticoduodenectomy (PD) is one of the most challenging operations with a high rate of complications and a measurable mortality risk even in high-volume hospitals [1–3]. Over the last 10 years, enhanced recovery programs such as fast track and enhanced recovery after surgery (ERAS) have been developed and applied to different branches of surgery. These programs involve a multimodal, evidence-based approach aimed at reducing surgical and anesthesiological stress and maintaining postoperative physiological function [4, 5]. Such approaches rely on a multidisciplinary team and individualization of all aspects of operative and perioperative care, including the use of minimally invasive techniques, optimal pain control, and postoperative rehabilitation including nutritional support and ambulation [5–8].

In 2012, the ERAS society proposed guidelines for perioperative care of patients undergoing PD [9] in this setting. However, the role of enhanced recovery protocols (ERp) in pancreatic surgery is still unclear, with only a single randomized trial having concluded that ERp can improve postoperative recovery of PD patients and reduce waiting time to chemotherapy [10].

Moreover, despite some initial success with ERp, studies have shown that the failure rate of items related to the patient is high and can involve more than half of patients in some instances. [11–15]. To date, the reported literature describes a scenario in which the application of an ERp after PD seems to be useful overall, even though many patients are unable to follow at least part of the protocol. The inability to comply with an ERp after PD is a real but an understated issue and better understanding of this limitation could help more patients benefit from an ERp. Here, we present our experience with ERp in pancreatic surgery with the aim of better understanding the causes of failure and identifying those patients who will be unable or unwilling to comply with an ERp.

## Materials and methods

This was a cohort study conducted on a hospital population in a single center. Data were collected prospectively in a predefined database. All patients provided written informed consent, and the study was approved by the local ethics committee.

The ERp utilized in our center consists of the following items: preoperative multidisciplinary information on the patient; no prolonged fasting before surgery (6 h fasting for solids, 3 h for fluids); antithrombotic prophylaxis with low molecular weight heparin (LMWH); no preoperative enema; specific short-term perioperative antibiotic prophylaxis; active intraoperative patient warming; postoperative nausea and vomiting (PONV) prophylaxis; individually tailored intraoperative fluid administration according to a goal-directed fluid therapy approach; continuous postoperative anesthetic wound infiltration for pain control to spare systemic opioids preferred to epidural analgesia; and standardized drain positioning and management. Other postoperative items are: nasogastric tube (NGT) removal on postoperative day (POD) 0; fluid introduction and the patient spending at least 4 h out of bed, with active ambulation, on POD 1; bladder catheter removal with the patient out of bed for at least 4 h, with active ambulation on POD 2; introduction of a solid diet on POD 3; and IV infusions stopped on POD 4.

### Operative technique

All surgical procedures were performed by experienced pancreatic surgeons with an open approach. Pylorus-preserving PD was routinely performed when oncologically appropriate and an end-to-side duodenojejunostomy was realized 30 cm distal to the biliary-enteric anastomosis. A double-layered, end-to-side, pancreatojejunostomy (preferable duct-to-mucosa) with either Child or Roux-en-Y technique was used for reconstruction.

### Collected variables

Perioperative data including all ERp items were prospectively collected in a database. The predictive score refers to a published risk score for postoperative complications specific for PD proposed by Braga et al. [16]. Pancreatic fistula, delayed gastric empting, and their grade were defined according to the ISGPS criteria [17, 18]. Postoperative complications were graded according to the Clavien-Dindo classification and major complications were defined as grade 3 or higher as is commonly accepted [19]. Antithrombotic and antibiotic prophylaxis were administered according to internal protocols at our center in accordance with international guidelines.

### Definition of ERp failure

There is no available definition of ERp failure. Previous reports have been more focused on assessing safety and feasibility of ERp than compliance with specific protocol items. None to date have focused on the overall ability of patients to meet the proposed postoperative items.

To define ERp failure, we focused on those items that were associated with a higher risk of failure both in our cohort and in previous reports [20]. Interestingly, these were primarily patient-related characteristics including mobilization, oral feeding, removal of bladder catheter, removal of NGT, and stopping of intravenous (IV) infusion. Failure to comply with individual aspects of the ERp were defined as: no active ambulation on POD 1; removal of NGT after POD 1; less than 4 h out of bed on POD 2 (mobilization); removal of bladder catheter after POD 3; reintroduction or suspension (for at least 1 day) of oral feeding after POD 4; and continuation or reintroduction of intravenous infusion after POD 4.

No previous work has assessed which and/or how many items of this program need to be failed to define a failure of the overall program. Intuitively, the inability of a patient to comply with a single item, e.g., removal of the bladder catheter by POD3, should not be considered sufficient to constitute overall failure of a complex, multimodal, perioperative management strategy. Conversely, a patient that is unable to achieve the majority of scheduled postoperative goals can hardly be considered as successfully treated according to the ERp. We assigned one point for each item failed and summed these to obtain an overall score. We then performed a correlation analysis between this number and postoperative length of stay and also evaluated its relation with postoperative complications. This analysis was used to define the numeric threshold of individual items failed in order to define the overall failure of the ERp.

### Creation of a predictive model

We developed a predictive model based on only pre- and intraoperative variables that are known to be clinically relevant for postoperative outcomes. Preoperative and intraoperative data that were included were age, body mass index (BMI), weight loss, ASA score, preoperative hemoglobin, use of preoperative chemotherapy, the presence of biliary stent, operative time, intraoperative blood loss, pancreatic consistency and Wirsung’s duct diameter. All of these parameters have been shown to correlate with postoperative outcomes [4, 17, 18, 21].

We defined our model by beginning with all these variables included and then reducing the number of variables included in the model following a backward and forward selection method, using the standard significance level for testing of hypotheses (*α* = 0.20) as stopping rule. An evaluation of the model with residuals, leverage points, and Cook distance analysis was performed.

The variation of each variable in the final model, predicting ERp failure, was attributed to a whole-number score based on logistic regression coefficient. The regression coefficient was calculated as following: ten units variation for age with the variable centered at 40 years, 60-min variation for operative time centered at 360 min and one unit variation for BMI centered at 25 kg/m^2^, and 10 points for a soft pancreas.

### Statistical analysis

Data were analyzed utilizing STATA 14.2. Data are presented as percentage, median with interquartile range [IQR], or mean with standard deviation (SD). *χ*^2^ test, *t* test, and logistic regression models were used to assess the association between different variables when indicated. The probability of failure of the protocol was modeled using logistic regression. To develop the model, we assessed the number of degrees of freedom granted by our patient population on the basis of the number of failures observed. Only the variables already known to be associated with postoperative outcomes were included in the model, and these were reduced while modeling using a different approach. An evaluation of the model with residuals, leverage points and Cook distance analysis and a sensitivity analysis were performed, excluding the identified cases, without observing any major variation in the model. Finally, we tried to translate the model into a practical clinical score using the coefficients of the logistic regression. With a sensitivity analysis we attributed a score from regression coefficients to each variable.

### ERp failure definition

The achievement and failure rate of ERp items are summarized in Table 1. The median number of failed items in our population was two with 153 (74.6%) of patients failing at least one item and 116 (56.6%) failing at least two items. Two failed items represent the median value in our population and could be considered an unbiased cutoff.

**Table 1:** ERp items

	Number (%)	Mean SD	Median IQR
Counseling preop	166 (80.1%)		
Avoid bowel preparation	205 (100%)		
Antibiotic prophylaxis	205 (100%)		
PONV prophylaxis	199 (98%)		
Antithrombotic prophylaxis	200 (97.6%)		
Patient’s warming	205 (100%)		
Prokinetics use	197 (97%)		
Fail NGT removal	9 (4.4%)		
POD fluid intake		1.4 [0.9]	1 [1]
POD solid intake		4 [1.9]	3 [1]
Fail solid oral intake	71 (34.6%)		
Fail stop ev infusions	102 (49.8)		
POD bladder catheter removal		3.6 [1.6]	3 [1]
Fail bladder catheter removal	83 (40.5%)		
NGT reinsertion	40 (19.5%)		
POD2 mobilization hours		3.4 [1.5]	4 [2]
Fail mobilization	82 (40%)		
POD2 deambulation meters		3.7 [6.6]	2 [0]
Fail deambulation	42 (20.5%)		
Fail one item	153 (74.6%)		
Fail two items	116 (56.6%)		
Number of items failed		1.9 [1.5]	2 [3]

Data are presented as number of patients (%), median with interquartile range [IQR] or mean with standard deviation (SD) *PONV* postoperative nausea and vomiting, *NGT* nasogastric tube, *POD* postoperative day

**Fig. 1:**
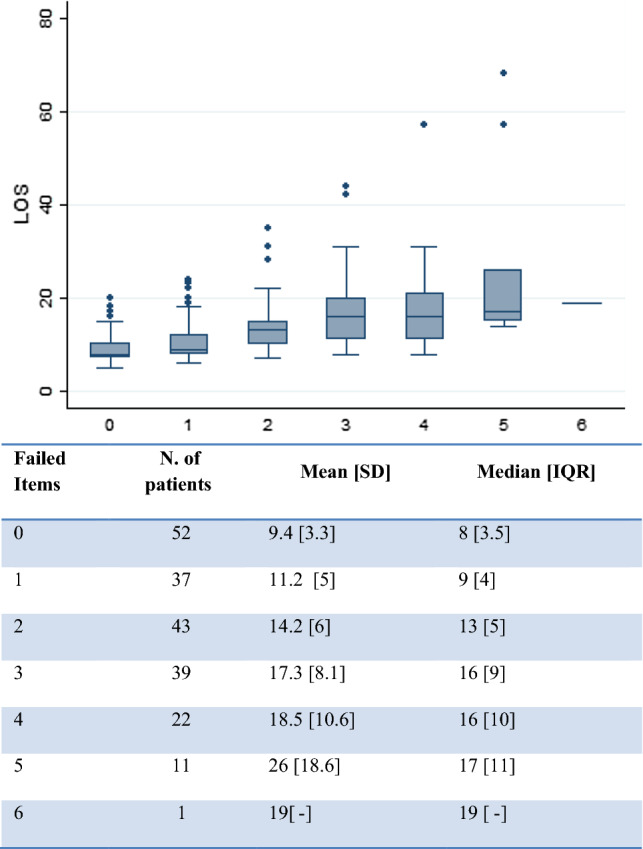
Box plot of the increase in length of stay at the increased number of items failed

Moreover, there was a progressive prolonging of the postoperative length of hospital stay with increasing sum of failed items (Fig. 1) [*χ*^2^(5) = 94.81; *p* < 0.001]. Postoperative length of stay is a crude but robust and broadly used indicator for patient recovery. The failure of at least two items identified patients with a median postoperative length of stay longer than our predefined goal of 10 days which was based on our historical control [22].

We then identified the failure of the protocol as the inability of the patient to comply with two or more items. This “a priori” definition of failure was utilized as the outcome for our predictive model.

## Results

A total of 205 consecutive patients underwent PD in our center and were managed according to an ERp between 1 January 2014 and 31 January 2016. Demographic and preoperative characteristics of the population are summarized in Table 2 and postoperative outcomes are reported in Table 3.

**Table 2:** Patient characteristics

	Number (%)	Mean SD	Median IQR
Male	119 (58%)		
Age		64.7 [13.7]	66 [19]
Age ≥80 years	18 (8.8%)		
Weight (kg)		68.2 [11.7]	69 [17]
Height (cm)		167 [14.4]	168 [15]
BMI		24.05 [3.9]	24.16 [4.8]
ASA score ≥3	37 (18%)		
Preoperative Hb		13 [2.2]	13.1 [2.4]
Preoperative Bilirubinemia		3 [5.4]	1 [1.9]
Diabetes	45 (22%)		
Insulin therapy	23 (11.2%)		
Preoperative biliary stenting	107 (52.2%)		
Malignant disease	179 (87.3%)		
Preoperative chemotherapy	11 (5.4%)		
Soft pancreas	68 (33.2%)		
Wirsung diameter (mm)		4.1 [2.1]	4 [2]
Roux-en-Y reconstruction	101 (49.3%)		
Vascular resection	11 (5.4%)		
Intraoperative transfusions (yes/mL)	77 (37.6%)	280.2 [668]	0 [500]
Blood loss (mL)		537.9 [604.5]	400 [350]
Blood loss ≥700	33 (16.1%)		
Predictive score		4.5 [3]	4 [4]

Data are presented as number of patients (%), median with interquartile range [IQR] or mean with standard deviation (SD) *ASA* American Society of Anesthesiologists, *BMI* body mass index

**Table 3:** Postoperative outcomes

	Number (%)	Mean SD	Median IQR
Pancreatic fistula (PF)	78 (38%)		
PF grade B/C	45 (22%)		
Biliary fistula	11 (5.4%)		
DGE	28 (13.7%)		
Respiratory complications	6 (2.9%)		
Infective complications	63 (30.7%)		
Postoperative transfusions	46 (22.4%)		
Overall complications	112 (54.6%)		
Major complications	32 (15.6%)		
Reinterventions	10 (4.9%)		
Readmissions	7 (3.4%)		
Mortality	2 (1%)		
POD fit for discharge		13 [8.3]	11 [9]
Length of hospital stay		14.1 [8.6]	12 [9]

Data are presented as number of patients (%), median with interquartile range [IQR] or mean with standard deviation (SD) *PF* pancreatic fistula, *DGE* delayed gastric emptying *POD* postoperative day

### ERp failure and postoperative complications

We identified the failure of the protocol as the inability of the patient to comply with two or more items. This “a priori” definition of failure was utilized as the outcome for our predictive model.

Interestingly, but unsurprisingly, the sum of failed items correlated with the occurrence of both overall and major postoperative complications. Patients without complications had a mean score of 1.13 (CI 0.88–1.37) versus a mean score of 2.53 (CI 2.25–2.81) for patients with complications (*p* < 0.001, two-sample t test). Similarly, patients without major complications had a mean score of 1.66 (CI 1.45–1.88) versus a mean score of 3.15 (CI 2.64–3.67) for patients with major complications (*p* = 0.001, two-sample *t*-test).

### Predictive model

A model that included the four variables of age, operative time, BMI and soft pancreas, with a good sensitivity of 80% and with a Hosmer–Lemeshow goodness to-fit test with *p* = 0.938 was obtained (Table 4). Each of age, operative time, BMI and soft pancreas were independent predictors of failure (OR 1.03 [1.001–1.06] *p* = 0.01; OR 1.11 [1.01–1.22] *p* = 0.03; OR 1.004 [1.001–1.009] *p* = 0.02 and OR 2.89 [1.48–5.67] *p* = 0.002, respectively).

**Table 4:** Predictive model of ERp failure

Variable	*p*	OR	CI 95%
Age (years)	0.01	1.03	1.001–1.06
BMI	0.03	1.11	1.009–1.22
Operative time (min)	0.02	1.01	1.001–1.01
Soft pancreas	0.002	2.89	1.49–5.67

We also provided a definition of ERp failure before modeling, based on analysis of the study population. To adjust for this approach, we performed a sensitivity analysis testing the ability of the model to predict an ERp failure defined by different cutoffs. When failure was defined as one or more failed items, we obtained a Hosmer–Lemeshow goodness to-fit statistic with *p* = 0.718; when the cutoff was three failed items, the *p*-value was 0.35; when it was four failed items, the *p*-value was 0.15.

The variation of each variable predicting ERp failure was attributed to a whole-number score based on logistic regression coefficient. The resulting score for each patient was used as a new variable on which we performed a ROC analysis, with the resulting area under the curve score of 0.747 [0.678–0.816]. A total of 75% of patients with a score of 14 or less points failed the program, and this score correlated with length of hospital stay (*r* = 0.52 [0.33–0.71]; *p* < 0.001). In order to better understand the correlation between ERp and postoperative complications, we also evaluated the correlation between the fistula risk score (FRS) [23], and the failure of ERp (AURC = 0.696).

## Discussion

Our study is the first attempt to specifically analyze the failure of an ERp in pancreatic surgery and understand if specific variables are correlated with this failure. Our results confirm previous observations [15, 16], and underline the problem of adherence to ERps after major surgical procedures. Previous literature shows that high compliance to aspects of the ERp that are based on patient management and the use of specific therapies and devices is achievable with good organization and the commitment of surgeons, anesthesiologists and nurses. Instead, failure rate is mainly high for those items in which the will and physical condition of the patient play a fundamental role, such as postoperative mobilization and reintroduction of oral feeding.

Surgical procedures on the pancreas are usually longer, more stressful for patients and with a higher rate of postoperative complications [21] than other types of surgery in which ERp has been applied. These observations may partly explain why the problem of protocol adherence has not been observed to this magnitude in other branches of surgery.

The number and rate of failed items after a PD was extremely high in our cohort. This was despite the significant experience of the group in ERp and pancreatic surgery, with around 100 PDs performed each year in our center. In 2010, an ERp for PD was introduced for the first time in our center and was fully implemented in July 2013. To avoid any learning-curve related bias, our analysis only included patients from 2014 onwards. Interestingly, the majority of failed items were postoperative and in most instances were patient-related. Thus, it was not unexpected to find variables, such as age and BMI, both of which are indicators of the patient’s physical status, included in the final predictive model. In addition, pancreatic softness was also included as a variable in the model. This may be because pancreatic stump texture is one of the main risk factors for the development of postoperative pancreatic fistula (POPF) [21], which are the main determinant of postoperative morbidity and mortality after pancreatic surgery [23], and so clearly have an impact on the recovery of the patient. In light of this evidence, it was theorized that use of the FRS [21] might partially predict ERp failure but the predictive power of this was found to be lower than using our model.

These two observations underline how patient ‘frailty’ and postoperative complications interact with the application of an ERp and its results. ERp has been studied to help improve the postoperative recovery of patients and to reduce complications. However, at the same time, the occurrence of postoperative complications can impair the ability of the patient to fulfill the goals of an ERp. The causal relationship between postoperative complications and inability to fulfill an ERp is unclear and will most likely remain an unsolved problem. The aim of the present study was not to further elucidate this complex relationship, but to demonstrate that is possible to identify the population of patients unlikely to fully follow the ERp and suggest an instrument to do so in everyday practice.

This work has some intrinsic weak points. Definition of our primary outcome, failure of the ERp, was based on analysis of our population data due to lack of ‘a priori’ definition in literature. Since this was the first attempt at defining and analyzing the problem of adherence to ERp after PD, it was necessary to create our own definitions. For this reason, we performed a sensitivity analysis demonstrating that even with varying definition cutoffs, the predictive ability of the model stands. However, external validation of the model in other datasets is required.

Nearly half of the patients who underwent PD failed to comply with our ERp and we strongly believe that the ability to predict ERp failure is of clinical relevance. For this last aim, we translated the model into a simple, easy-to-use scoring system that can be employed in standard clinical practice. The creation of a prognostic score to predict the failure of ERp is the first step in trying to extend the benefit of ERps to the whole patient population. The score could probably be improved with the introduction of new variables that better define relevant patient characteristics (e.g., the frailty index) and preoperative comorbidity in order to identify patients at risk of postoperative complications.

A better characterization of the subpopulation that do not manage to follow the ERp and a strategy to reduce failure rates should represent the next goal for groups that believe in the usefulness of ERp. Tailoring the ERp to the individual patient may be one approach to improve postoperative recovery, especially in fragile and complicated patients that will otherwise simply fail to comply with objectives too burdensome for them. In order to understand what might be the best solution for this underrated problem, the first step is to analyze and demonstrate that is possible to identify characteristics that correlate with the failure of ERp. Our work, despite its limitations, represents the first attempt to address this question.

If this issue is not addressed, efforts to implement and ameliorate ERps will only offer a clear benefit to around half of our patients, with any advantage to the remaining half non-measurable and perhaps nonexistent.

## Conclusions

Starting from pre- and intraoperative data, we identified a subpopulation of patients at high risk of no compliance to ERp. By investing further effort in this particular subpopulation of patients, we may be able to increase the efficacy of ERp after pancreatic surgery and expand its use in clinical practice.
